# Preventable perinatal deaths in indigenous Wixárika communities: an ethnographic study of pregnancy, childbirth and structural violence

**DOI:** 10.1186/s12884-018-1870-6

**Published:** 2018-06-18

**Authors:** Jennie Gamlin, Seth Holmes

**Affiliations:** 1 University College London Institute for Global Health, London, UK; 2 University of California, Berkeley, USA

**Keywords:** Perinatal mortality, Indigenous, Structural violence, Childbirth

## Abstract

**Background:**

Preventable maternal and infant mortality continues to be significantly higher in Latin American indigenous regions compared to non-indigenous, with inequalities of race, gender and poverty exacerbated by deficiencies in service provision. Standard programmes aimed at improving perinatal health have had a limited impact on mortality rates in these populations, and state and national statistical data and evaluations of services are of little relevance to the environments that most indigenous ethnicities inhabit. This study sought a novel perspective on causes and solutions by considering how structural, cultural and relational factors intersect to make indigenous women and babies more vulnerable to morbidity and mortality.

**Methods:**

We explored how structural inequalities and interpersonal relationships impact decision-making about care seeking during pregnancy and childbirth in Wixarika communities in Northwestern Mexico. Sixty-two women were interviewed while pregnant and followed-up after the birth of their child. Observational data was collected over 18 months, producing more than five hundred pages of field notes.

**Results:**

Of the 62 women interviewed, 33 gave birth at home without skilled attendance, including 5 who delivered completely alone. Five babies died during labour or shortly thereafter, we present here 3 of these events as case studies. We identified that the structure of service provision, in which providers have several contiguous days off, combined with a poor patient-provider dynamic and the sometimes non-consensual imposition of biomedical practices acted as deterrents to institutional delivery. Data also suggested that men have important roles to play supporting their partners during labour and birth.

**Conclusions:**

Stillbirths and neonatal deaths occurring in a context of unnecessary lone and unassisted deliveries are structurally generated forms of violence: preventable morbidities or mortalities that are the result of systematic inequalities and health system weaknesses. These results counter the common assumption that the choices of indigenous women to avoid institutional delivery are irrational, cultural or due to a lack of education. Rather, our data indicate that institutional arrangements and interpersonal interactions in the health system contribute to preventable deaths. Addressing these issues requires important, but achievable, changes in service provision and resource allocation in addition to long term, culturally-appropriate strategies.

**Electronic supplementary material:**

The online version of this article (10.1186/s12884-018-1870-6) contains supplementary material, which is available to authorized users.

## Background

The Wixárika ethnic group of Northwestern Mexico is accustomed to high rates of maternal and infant mortality, and until the 1970s they had no vehicle access, schools or medical care of any kind. Although today their most accessible villages can be reached within 2–5 h by unpaved track, approximately half of their population of approximately 35,000 continue to live a further six to 8 h away on foot [1–3]. As we demonstrate and discuss below, distance from medical facilities is only one of the structural factors that continue to lead to high rates of perinatal mortality in their communities.

Maternal and infant mortality rates are unacceptably high in Mexico’s indigenous regions compared to non-indigenous, with the largest proportion of mortality now occurring in the perinatal period [4]. This reflects a global trend, neonatal mortality accounted for 42% of under-five mortality in 2013, an increase from 37% in 1990 [5]. Using the most recent comparative data, the Mexican Infant Mortality Rate (IMR) was 14 per thousand live births in the non-indigenous population, yet 23 in indigenous populations [6]. Although the national under-five mortality rate (U5MR) is now only slightly above the fourth Millennium Development Goal target of 15 per thousand [7], Mexican indigenous people fare far worse. The U5MR was 76 [8] in the indigenous municipality of Mezquitic where this study was conducted (there were no municipal data available on perinatal, neonatal or infant mortality), reflecting the global tendency for worse health outcomes among indigenous people in contrast to non-indigenous people within the same nations [9–11].

Lack of skilled birth attendance is one of the major causes of infant mortality in indigenous regions, with only 76% of indigenous compared to 94% of non-indigenous women delivering in health facilities [12]. Although 93% of births in Mexico were assisted by a skilled birth attendant (SBA) in 2014, only 30% of births to women in the indigenous muncipality of Mezquitic were attended by a SBA and an estimated 55% were home births [13]. Unlike some southern Mexican indigenous groups, it is known that the Wixárika do not have a culture of traditional midwifery, and that women who birth at home often do so alone [3, 14]. This is also the case with the Rarámuri, who live further north in the same mountain range, and is likely due to the spatial organisation of living arrangements [15]. Both ethnic groups traditionally live in extended family ranches at considerable distance from each other, and if not alone, women may be assisted during birth by a family member or mará’kame (shaman) [15].

A recently published systematic review suggests that skilled birth attendance could reduce neonatal mortality by 25% [16]. Although in theory Mexico now has universal health coverage through the *Seguro Popular* (SP) health programme and enrolment in public insurance programmes increased from 55 million to almost 120 million between 2004 and 2011 [17], there has not been a concomitant increase in service provision nor financial resources [18] and there remain serious structural barriers to accessing maternal health services. In Mexico’s indigenous regions, these include anti-indigenous discrimination, quality of care, gender inequality, language and cultural barriers, poverty, mistreatment and abuse as well as physical distance and means of communication [19–21].

Considerable ethnographic research has documented the role of *structural violence* in maternal deaths among indigenous women, where the intersections of gender inequality, ethnic hierarchies and poverty compound the problem of access to health facilities [22–25]. Structural violence is an umbrella term used by medical social scientists to denote the harm done by social, political and economic structures and institutions, or “social arrangements that put individuals and populations in harm’s way” ([26], p1686). The concept of ‘everyday violence’, denotes the normalised or daily (‘everyday’) and therefore unnoticed occurrence of structural inequalities leading to increased morbidity and mortality – or structural violence. Throughout her work documenting the ‘violence of everyday life’ in in a Brazilian shantytown, Scheper-Hughes discusses how infant deaths, forms of everyday violence, were ‘generally thought of as dispensable, as hardly worth counting at all’ ([27] p216). Here, we seek not to focus solely on individual actions or inactions to explain morbidity and mortality in a context of poverty, but rather to link experiences of sickness and death to the social, economic and political conditions that structure the risk of people in different social positions. While Mexico has seen a reduction in maternal and perinatal mortality over the past decade, this decline has been considerably weaker and has stagnated in indigenous regions [28, 29]. In this paper, we take a structural violence approach to exploring this inequality by focussing on the ways in which maternal and perinatal mortality track along structural hierarchies, with correlated lack of skilled birth attendance leading to increased mortality risk for both mother and baby. We share in- depth interview and ethnographic observation data on births, deaths, and maternal experiences in clinics and hospitals. We analyse this ethnographic case data alongside contextual data in order to understand how structural violence operates in both interpersonal relationships and the structure of health services provision to put indigenous women and their babies at risk during childbirth and the perinatal period.

## Methods

This study aimed to explore the structural determinants of maternal and perinatal health in a Wixárika community located in Northwestern Mexico. Our objective was to understand women’s experiences of care in clinics and hospitals and how these relate to decreased rates of institutional delivery.

### Setting

Our study took place in the towns and villages that make up the indigenous governorship of Yuáwime (not real name), with a population of approximately 2500. Spread over three highland governorships, 77% of the municipality’s population of 18,084 are indigenous [13]. About half of the population of Yuáwime live in one of two vehicle-accessible towns located at up to 5 h from the nearest hospital, the other half live in hamlets up to a further 6 h walking distance. Covering 767 km^2^, the governorship is divided by a deep valley between the two towns, each of which has a rural health clinic (URM: *Unidad Medica Rural*). Officially, all highland clinics under the same medical jurisdiction are staffed from the 11th to the 30th of each month by a general practitioner, a nurse, and a travelling doctor who visits villages on foot. In addition, a student doctor attends the clinic from the 21st of each month for 20 days, running it alone from days 1 to 11.

### Design

Anthropological studies including gaining community trust, gathering birth stories and verbal autopsies are recognised as important strategies for research with indigenous populations in settings where epidemiological data are lacking, unreliable or very difficult to obtain [30]. This broad ethnographic study included interviews with women while they were pregnant and after delivery as well as long-term observations of family and community dynamics. During pregnancy we asked about socio-demographics, gender equality, previous pregnancies, antenatal care, and delivery intentions. Postpartum interviews were semi-structured and used a checklist of questions about birth attendance, outcomes and experiences (Additional file 1). The interview guide included prompts for women to report on choices regarding delivery, treatment by health professionals, and institutional care. Observations were performed and field notes collected with pregnant women and the wider community. We report on the findings of these data according to the Consolidated Criteria for Reporting Qualitative studies, COREQ [31].

### Research team and reflexivity

The principal investigator, who conducted all observations, is a medical anthropologist with expertise in health inequalities. She had worked in Yuáwime since 2008. Eight female bilingual (Wixárika/Spanish) women were trained as interviewers using pilot questionnaires with non-study women, and the forward-back translation method was used to ensure shared understanding in Wixárika or Spanish. The interviewers were all residents of Yuáwime and worked part-time on the study over the course of 2 years. Postpartum interviews were conducted by the principal investigator with translation assistance.

### Procedures

Interviews and observations were conducted as part of a wider ethnographic study of the structural determinants of maternal and child health in Wixárika communities. We used ethnographic methods to explore the structural origins of maternal morbidity and mortality by analysing interactions among people and between people and institutions. The study was granted ethical approval by University College London Ethics committee and the governorship’s General Assembly meeting. Interviewees consented verbally according to these approvals and all people’s names and place’s names have been changed for anonymity.

### Participant selection

All women who were known to be pregnant and could be contacted were invited to participate in the study at community meetings. Interviewers then made visits to the homes of pregnant women, who had been identified by word of mouth, to extend a personal invitation. Sixty-seven women were interviewed at baseline and there were eight refusals, with most citing a reluctance to share personal information. We were interested in all women who were pregnant, but due to the practical difficulties of finding women from distant valley communities in their homes, we expect that this group are underrepresented in our sample.

### Data collection

The pregnancy interview asked participants about antenatal care, birthing intentions, fertility history and household division of tasks. Interviewers were encouraged to use their local knowledge to conduct interviews when the husband or other male relatives were not present. Women were followed up in their homes after delivery with audio-recorded semi-structured interviews lasting between 15 min and 1 h. The 2-year process was supported by observational data recorded in field notes, and informal interviews with health providers, community elders and traditional healers for data triangulation. Interviews were transcribed directly into Spanish and findings were presented to women at later community meetings and a public engagement event.

### Data analysis

Numerical data were extracted from baseline and in-depth interviews to generate descriptive data on parity, age and birthing intentions and pregnancy outcomes. A similar process of extracting numerical data from ethnographic research has been used by anthropologists Scheper-Hughes and Briggs to investigate perinatal deaths where accurate data was not previously available [27, 32]. We conducted an inductive thematic analysis of all qualitative data on N-Vivo (version 8). Following the general practice of grounded theory [33], data that was coded together included interview transcripts and observational field notes. We first identified general codes (themes) and then added sub-codes to each theme. Finally codes were collapsed into a limited number of themes for the purpose of writing. Data were coded separately in Spanish by one bilingual (Wixárika-Spanish) and one Spanish-speaking research assistant and checked for accuracy by a third scholar. Emergent themes included social and cultural issues related to maternal and child health, Wixárika traditions and customs, gender inequalities, and health systems issues. Within the wider ethnographic dataset, we focus here especially on pseudonymised information on health systems and interpersonal relationships. We focus on both community-wide data and on in-depth ethnographic case descriptions in order to link individual experiences with larger social structures.

## Results

### Clinic services

Most women participants lived in extended families in their highland communities. Basic healthcare was provided from each of the two clinics (URM), whose doctors lived on–site. Although both clinics worked to the same regulations, there were important differences in their staffing and facilities. The clinic in community “A” had one examination room with a gynaecological bed. Providers and clients had to walk through the consultation area to enter and leave the clinic, use the toilet, or access the examination room. Clinic “B” had been reconstructed recently in line with Ministry of Health requirements and had two separate consultation rooms, a birthing room equipped with gynaecological bed, and a recovery room.

Both clinics were clean but sparsely equipped; each stocked basic medications for common ailments and problems specific to the region, including scorpion antivenom. Biological samples were not taken at either clinic as they could not be stored or transported in a safe or timely manner: these were carried out at the regional hospital located 3–5 h away by bus. According to official guidelines, URM staff should not attend births, which should be channelled to the nearest hospital. In practice, appreciable numbers of births took place in the highland clinics. There was a daily bus service from community “B” and a twice-weekly service from community “A” to the town where the nearest hospital was located. A return bus journey cost between US$15–20, approximately 20% of a family’s monthly income. An overnight hospital stay would incur additional costs for food, bus fares for other family members, and in some cases hotel accommodation. Women with high-risk pregnancies were referred to hospital and could stay in municipal hostels for the final weeks of pregnancy, although only one woman in our sample chose this option. Each URM has an ambulance used strictly for emergencies.

### Pregnancies and outcomes

Sixty-seven baseline interviews were carried out with women of mean age 24.5 years (range 13–39), 17 of whom were primigravid (Table 1). Multigravid women had a mean 3.1 children (range 1–9) at the time of first interview. Based on birth data from Mexico’s National Institute on Geography and Statistics (INEGI) we estimate our sample to represent approximately 35% of births to women residents in Yuáwime during the data collection period [13].

**Table 1:** Details of birth location and attendance, based on 62 interview pairs

	URM A	URM B	Mean age of mother	Primigravidae	Mean previous live births
Lone birth	2	3	27.2	0	3.8
Home birth with unskilled help	14	14	28.3	5	3.5
URM birth	4	17	23.4	7	2.09
Hospital birth	3	5	23.1	3	2.14

Five women lost their babies at term, during or shortly after birth (Table 2). None of these five babies had a death certificate at the time of interview. The classification of stillbirth and neonatal death in Table 2 is based on birth narratives provided by mothers during interview. We present representative case studies of three of these deaths to flesh out the antecedents to the deaths beyond what can be ascertained by survey data alone.

**Table 2:** Perinatal mortality: Cases of stillbirth and neonatal death

Case numbers	Place of birth	Closest URM	Parity	Stillbirth or neonatal death
i	Home	B	5	Stillbirth
ii	Home	B	1	Stillbirth
iii	Home	A	1	Neonatal death
iv	URM	B	1	Neonatal death
v	Hospital	B	2	Stillbirth

### Case studies of infant deaths

i. Kupaima’s baby was born in Ximeri, one of the most distant communities, 5 to 7 h walk down into the valley from the nearest health clinic in town “B”. The birth narrative was told by a female family member who had been present during the birth.
  ‘Yes, we were here [in town “B”] for more than a month waiting for the baby to be born. We were here for the whole of the month, then we went back to their distant home community several weeks later with the intention of coming back soon, but we had to stay because I owed money […]. So we said “lets wait until I pay the money, then go”, and it was in those days that the pains started. […] I didn’t know what to do, I was in shock because I was alone. I sent a family member for the shaman who came and checked her, but couldn’t do anything. It’s because the baby was born feet first, that’s why it died. I pulled its foot, but it was already floppy, its Mum nearly died too. The baby died because he was born feet first, he was fine but he got stuck and he died; I had to pull him out.
  [Before we left for our home community,] I said to her husband he should look after her, then he went telling us that he would be back before the end of the month, but he didn’t return. When he arrived I said to him “take her to the clinic so that they give her something, she has lost a lot of blood”. But he didn’t take any notice of me. He just said, “they will tell us off”. She was in bed for a month. In that whole month she only saw the shaman. She would go unconscious for moments, we nearly lost her…’.
ii. Tutu’s baby was born in a house by the road, on the way to clinic “B”. Her birth narrative is told by one of her female relatives, who was present with her at the time.
  ‘Tutu’s baby was born in the holidays, in Juana’s kitchen. Juana’s husband is a shaman, that’s why women go there. But she took a long time, about 3 h, I just listened to her screaming and screaming; afterwards they took her to the clinic, but the baby was already dead.
  She didn’t go to the clinic because she thought the doctors weren’t going to be there. It was the day after they paid us [the day before the doctor leaves that community for vacation each month]. She had been in pain for 3 days, then she came up with the payment, she was going to go to the hospital but she couldn’t get a ride, so she stayed here and her baby came in the kitchen. She is still too sad to talk about it.
iii. Cecilia’s baby was born in a highland clinic. The birth was uncomplicated, but the baby died 8 days later. A close female family member, who was present with her at the time, narrated the events that led to the baby’s death.
  ‘The doctors were there and she gave birth in the clinic…. The first 3 days he was fine, but then he became ill, he would become shocked, as if something was scaring him, so we took him to the shaman, but he didn’t get better. We just became very sad and didn’t know what to do. He became worse after the vaccination and when we took him to the shaman, he said that [baby] was very sick. Maybe we could have done something but we didn’t have any money, so we didn’t take him anywhere, [we thought] “what will we do if they send us far away” [to a city hospital]. Also in the last few days the baby looked really sick, like he wasn’t going to survive’.
  ‘Then when I asked her, she didn’t want to tell me who the father was. But when I insisted, for the third time, she told me that a man had forced her to have sex, and I asked her, “and what did he say to you when he saw you pregnant? Tell him to take you to the clinic to check you, to see how the baby is, and you know that I don’t have time for ceremonies and that you will have a hard time if you are alone when you are in labour”. She replied “since he abused me he has not spoken to me, I think he only did it to humiliate me and now I feel bad”. Then the baby was born and all this happened.’

### The structure of health provision affecting infant survival

#### Quality of care, mistreatment and abuse

Like Cecilia, women who had chosen to give birth at one of the URM were mostly satisfied with their treatment. They reported being attended to by the doctors on duty and spoken to considerately, although most were also given a routine episiotomy to which they did not consent or of which they were unaware. As Hautsima said, ‘When Leandro’s sister was born, they cut me, I felt like kicking them in’.

Women who gave birth in hospital were less satisfied and described verbal abuse and disrespectful treatment. Wenima recalled that the doctor attending her had shouted, ‘Don’t you understand? Are we talking to a person or a dog?’ While Ema, who was 14 when she gave birth to her first child, said that the doctors had continually asked her age and chided her: ‘What were you doing? Were you playing “Mummies and Daddies”?’ When she complained of pain, they intimidated her with the phrase ‘you didn’t complain when he gave it to you, why are you complaining now?’. All the women who gave birth in hospital reported early discharge, often at young ages and with preterm or small newborns. Etsiama gave birth at 7 months gestation and was discharged 8 h later, while Tukima was discharged the following day with a baby born at 8 months and with low birthweight. None of these practices match the regional standard of care. Other routine practices, such as insistence on a supine delivery and removal of clothes are not only culturally inappropriate (see Campos 2016 for further context on intercultural medical practices), but also medically unnecessary practices. Matilde, whose baby was born in clinic “A”, described the moment she arrived at the clinic for a check up. She said ‘[next] they took my clothes off me, they made me lie down and they gave me injections and a drip. I was really embarrassed, but what could I do?’

During the study, three different doctors were posted to clinic “A”, one of whom was accused of insisting on a series of measures that were found offensive and provocative by members of the community. Regina, a clinic staff member, said,

‘She told us she was in charge and we had to do what she said. We were told we had to make an appointment the day before, but it takes some women hours to walk to the clinic; then if we arrive late she won’t see us and we have to come back another day. She also told us we would lose our Prospera [welfare] money if they don’t come for the exercise sessions, even if they have to walk from Metseri or Ximeri [2-4 hours walking distance]’.

The case of this particular doctor led the community to submit a series of official complaints to the Ministry of Health, including a request for the doctor to be replaced.

#### Weaknesses in service provision leading to home births

Doctors in clinic A were hired on short-term contracts at a monthly salary of approximately USD $400. This was less than half the salary of a permanent position and they moved on ‘as soon as something better paid comes up’ (Nurse, clinic “A”). Student doctors were on 1-year placements and often abandoned the posting early. On two occasions, fights broke out between clinic staff and members of the community, adding to tensions with the community. For the first 6 months of the study, there was no family doctor in clinic “A” and from the 11th to 21st only a nurse and health promoter. With each change of staff, the clinic would lack either a family doctor or a student doctor for weeks or months, leaving the impression that there was “*never anyone there”*, a statement made on multiple occasions by many different women.

Concerns with the structure of service provision intersect with the problem of transport for emergencies. Clinic “A” was located at least 4 h on a dirt road from the nearest hospital and medical staff were concerned about the potential legal and political implications of maternal and perinatal death. Although there was more trust in the staff in clinic “B” and the drive to hospital was shorter, the ambulance could only be driven by the doctor or nurse. This meant that for the 10 days during which only a student doctor was on duty, the ambulance was not available.

Women who chose to deliver at home did so because they shared a deep scepticism, often based on experience, about the availability of staff and anticipated poor or invasive treatment. When asked why they chose a home birth, women spoke of fear. Marcela, simply said that ‘I didn’t go because I was scared they would cut me’, referencing the common practice of unconsented episiotomy. Others expressed embarrassment that spoke to feelings of humiliation at being told off, as Anahi indicated in her interview, ‘there was a doctor who told me off a lot, telling me I should do exercise. And now I thought they would say the same and I thought “better I don’t go”’.

The official narrative from medical professionals was that ‘there is always someone in the clinic’ (Nurse, clinic “A”). However, this was not observed by researchers and is not the experience of many interviewees. Everyone knew that for the first 10 days of each month, only a student staffed the clinic and many interpreted this as there ‘not being anyone there’. Haulima who gave birth to her second baby in her Aunt’s kitchen, said that she chose not to go to the clinic because ‘my son was born in the holidays and I thought the doctors wouldn’t be there… he was born in the first days of the month and they arrive on the 11th.’ This concern about not being received was coupled with a previous bad experience of delivery with her ankles ‘strapped high above her head’*.* Haulima was fortunate: her baby was born well, but her sister-in-law Tutu, whose birth is narrated above, lost her baby 2 months before in the same kitchen on the same day of the month.

There were major differences in service provision in the two URM. While in clinic “B”, the doctor had gained considerable trust in the community and women were confident that she would receive them between the 11th and 30th of each month, clinic “A” had a frequent turnover of doctors and was often unstaffed or had only a nurse on duty. An unwillingness to conduct deliveries was shared by all of the staff in clinic “A”. A nurse said, ‘If a women comes in labour I tell her to go; we don’t attend births here unless we absolutely have to*,*’ while the family doctor at the same clinic explained that she sent labouring women to the hospital 4 h away because ‘we often have no gloves, no oxytocin, no vitamin K and no serum.’

## Discussion

In this paper, we have explored indigenous women’s experiences of birth in a context characterised by profound historical structural inequalities, that are the consequence of their position as an ethnic minority group economically and socially marginalized within a relatively wealthy nation state (see for example Bonfil Batalla [34] or Batolomé [35] for historical accounts of the well-documented anti-indigenous discrimination and impoverishment in Mexico). Racial, economic and geographical marginalisation have intersected throughout history and, as our data show, today expose women and babies to structural violence in the form of preventable morbidity and mortality.

We acknowledge that the study is limited by our focus on depth over breadth. Ethnographic research about structural violence seeks to link broad structural inequalities to the local, micro-environment, including personal and group experiences of sickness, injury, and death. For this reason, we focus on a small regional population as opposed to a geographically broader and potentially more generalizable sample. A further limitation is that the geographical dispersion of the population is likely to have weighted our sample towards women living in more accessible locations who are more likely to have given birth in facilities, and, therefore, to underrepresent populations without skilled birth attendance. In addition, other social and economic factors including higher rates of seasonal migration among families in the most distant communities may exclude a higher proportion of marginalised families. These limitations suggest that our findings may underestimate the true proportion of women who give birth completely alone, and, therefore, also the extent of preventable deaths. This data vacuum warrants rigorous ethnographic and epidemiological studies with in-depth, implementation of verbal autopsy [27, 30] to identify causes of death where it has not been possible to establish this clinically.

In spite of these limitations, this study echoes public health and social science literature documenting physical distance and limited connectivity, cultural and language barriers, and extreme poverty as challenges to healthcare access faced by a majority of Mexican indigenous communities that also impacts perinatal and maternal mortality [36–38]. Analyses of the structural determinants of maternal mortality in the state of Chiapas highlight the role of racism, gender inequality and poverty in producing higher mortality among indigenous populations [24, 29]. An ethnographic study with indigenous communities in the state of Oaxaca, which together with Chiapas and Guerrero shares the double vulnerability of widespread extreme poverty and a high proportion of indigeneity [3], describes structural and gender violence, as well as mistreatment and abuse by healthcare providers operating as deterrents to service uptake [21–24]. These studies demonstrate how structural violence operates at the intersection of poverty, gender, and racial inequality to influence access to healthcare as well as morbidity and mortality. Considerable research also highlights more generally the need to address social exclusion and health inequalities in Mexican indigenous regions [12, 39, 40].

Our data extend structural analyses of gender, race, and poverty by exploring the ways in which the organization of service delivery adds an additional layer of structural violence. Our data show that the system of healthcare delivery may function to deter women from seeking skilled care during pregnancy and delivery with implications for perinatal mortality [41].

An in-depth ethnographic study in Oaxaca state reached similar conclusions about the unavailability of medical staff during clinic hours in indigenous communities [42].

The discrepancy between clinics suggests that higher quality of care is possible if there is continuity of clinic staff. Notwithstanding the limitation of our data, the numbers of women who delivered in clinic “A” (*n* = 4, 17%) where there was a high turnover of staff, compared to clinic “B” (*n* = 17, 44%) where there was greater confidence in the availability a doctor would appear to corroborate our ethnographic findings. In addition, women explained that the routine practices of unconsented episiotomy, supine delivery and insistence in removal of clothes were reasons for avoiding delivery in a health facility. These unnecessary interventions and practices, coupled with experiences of verbal abuse, the use of humiliating language and reprimanding of indigenous women were also cited as reasons for giving birth at home.

In addition to identifying how structural barriers to care lead to preventable mortality, our paper also elucidates gaps in care and interventions that could address some of these issues. Although not stated in these precise words by women, the lack of cultural competence and cultural humility on behalf of doctors and the absence of a system for dealing with obstetric emergencies that occur outside health facilities are evident failings in service provision. The intercultural medical practices proposed for work in Mexico indigenous communities [43] would greatly improve clinician-patient dynamics here. Women were told to travel to the hospital to avoid potential complications although there is not system in place to support them logistically or financially so that they are able to achieve this. A strategy named ‘Madrinas Obstéricas’ (Obstetric Godmothers) was in place in other states with the purpose of accompanying women to distant hospitals, as both social and cultural mediation support. Such a service could help bridge the gap between the doctor’s limited understanding of the realities and demands of Wixarika lives, women’s expressed desperation at not knowing what to do in case of emergencies and some of the barriers to accessing care that are faced by women who are either monolingual or speak only very limited Spanish.

However, infrequent bus availability and lack of funds for the bus would make even this intervention fail for many women. Thus, not only culturally appropriate and supportive projects like Madrinas Obstéricas, but also structurally responsive interventions would be needed. In many areas of medicine, “structural competency” is being taken on in order to address the health effects of structural violence [44, 45]. This approach within medical training consists of developing a core series of skills for working in setting of social and economic inequalities that affect both health outcomes and health care itself. Recognising the structures that shape clinical interactions, observing and imagining structural interventions, and re-articulating common frameworks for understanding health in structural terms are three of these skills that are of particular relevance to the perinatal care of indigenous women in Mexico, and could make an important contribution to improving clinical care.

Gender inequality impacts indigenous women’s care seeking decisions in Mexico, as has been demonstrated well [22–24]. Our data suggest also that men may have an important role to play in ensuring women have access to care, and must ultimately play a role in future interventions aimed at addressing gender inequality. In the current social context, men are better positioned to procure financial resources for transportation and are often required in practice for important decisions about care. Gender inequality is a structural barrier to healthcare that will require multiple culturally-appropriate strategies, first to increase the social and economic power of women and, second, to encourage male relatives to support healthy birth. Short-, medium- and long-term strategies must be developed to address this structural inequality.

A recent study of access to antenatal care in six Mesoamerican (Mexico and Central America) countries found that many women in the poorest regions were not receiving adequate antenatal care, [46] while a study of the Salud Mesoamérica initiative reported institutional delivery (with a skilled attendant) variation between 24% in Guatemala and 88% in Nicaragua [47], suggesting that our findings may be relevant to a wide geographical region.

## Conclusions

As a member of the Organisation for Economic Cooperation and Development (OECD) Mexico is not poor: it is rather very unequal. As it approaches universal health coverage, Mexico is in a position to improve care for marginalized groups in the short- to medium-term. A recent WHO press release describes investment in health employment as key to attaining the Sustainable Development Goals [48]; our findings suggest that investing in local service provision could bring important improvements to perinatal survival. Accurate vital registration will also be needed if we are to know whether the health system is working effectively.

Structural factors determine how and where women decide to give birth, why they choose unassisted births at home over health facility births, and ultimately how likely they are to experience perinatal deaths. While common frameworks for understanding maternal health in medicine and public health indicate that women must be educated to seek prenatal and obstetric care in a timely fashion, our research indicates that many indigenous women in Latin America have good reason for avoiding medical care. Thus, medical and public health interventions to improve childbirth and maternal survival rates must not focus only on the knowledge, beliefs and behaviours of mothers, but perhaps more importantly on the organization of health care, the attitudes and practices of health professionals in relation to indigenous women, and societal gender equity. These are important, achievable targets for a middle-income country.

## Additional file

Additional file 1: English language version interview guide. (DOCX 90 kb)

## Abbreviations

COREQ: Consolidated Criteria for Reporting on Qualitative Studies
IMR: Infant Mortality Rate
INEGI: Instituto Nacional de Estadistica e Geografia (National Institute for Statistics and Geography)
OECD: Organisation for Economic Cooperation and Development
OMM: Observatory for Maternal Mortality
SBA: Skilled Birth Attendant
SP: Seguro Popular
U5MR: Under 5 Mortality Rate
UMR: Unidad Médico Rural
WHO: World Health Organisation
