# Supplementary material for: Preventable perinatal deaths in indigenous Wixárika communities: an ethnographic study of pregnancy, childbirth and structural violence
Source: BMC Pregnancy Childbirth. 2018 Jun 18;18:243. doi: 10.1186/s12884-018-1870-6 (PMC6006582; doi:10.1186/s12884-018-1870-6)
Supplement: Supplementary file 1 — English language version interview guide. (DOCX 90 kb) [file 12884_2018_1870_MOESM1_ESM.docx]

Interview guide –question checklist

Request consent to interview [Set script in Wixárika]

Request consent to use recorder

Switch on recorder AFTER consent. If no consent to record, use hand written notes.

| **Main theme** | **Prompt** | **Prompt** | **Prompt** |
| --- | --- | --- | --- |
| **Birth narrative** | Name, age, place of origin |  |  |
|  | Location (home/clinic) | Location (Place name) | *Whose* home (parent, own, mara’akame, other) |
|  | Outcome (live/dead/stillbirth) |  |  |
|  | Complications |  |  |
| **If home birth** | Birth narrative |  |  |
|  | Position and progression of labour |  |  |
|  | Help/assistance (who) | How assisted |  |
|  | Mara’akame? |  |  |
|  | Complications? |  |  |
|  | Role of husband? (if there) |  |  |
| **Clinic/Institutional birth** | Birth narrative |  |  |
|  | Position and progression of labour |  |  |
|  | Doctor in attendance | How feel about doctor behaviour/  presence | How feel about institutional practices in general |
|  | Complications | How resolved? |  |
|  | Mara’akame? |  |  |
|  | Interventions? | Informed consent? | How feel about intervention? |
|  | Medication? | Informed consent? | How feel about medication? |
| **In case of death** | Cause? (In case of live birth) | If traditional causality, ask for background. | If not known, ask for visible symptoms. |
|  | Registration | Follow up with authorities? |  |
| **Future intentions** | Is there anything you would do differently next time? |  |  |
|  | Where would you like to give birth next time? |  |  |

**Thank participants for interview!**
